# Die Geschichte der kardialen Resynchronisationstherapie: 30 Jahre Elektrotherapie der Herzinsuffizienz

**DOI:** 10.1007/s00399-024-01004-2

**Published:** 2024-02-29

**Authors:** Christoph Stellbrink

**Affiliations:** Universitätsklinikum OWL Campus Klinikum Bielefeld., Universitätsklinik für Kardiologie und Internistische Intensivmedizin, Teutoburger Straße 50, 33604 Bielefeld, Deutschland

**Keywords:** Herzinsuffizienz, Linksschenkelblock, Herzschrittmacher, Kardiale Resynchronisation, Conduction System Pacing, Heart failure, Left bundle branch block, Pacemaker, Cardiac resynchronization therapy, Conduction System Pacing

## Abstract

Vor mehr als 30 Jahren erfolgte die erste Implantation eines permanenten, biventrikulären Herzschrittmachersystems bei einem Patienten mit Herzinsuffizienz und ventrikulärer Leitungsstörung. In diesem Artikel soll die historische Entwicklung der kardialen Resynchronisationstherapie (CRT) vom pathophysiologischen Konzept über die ersten *Proof of concept-Studien* bis hin zu den großen randomisierten Studien, die dann zum Einzug der CRT in die Leitlinien zur Behandlung der Herzinsuffizienz geführt haben, dargestellt werden. Auch nach der Etablierung der CRT kam es zum einen zu einer Ausweitung der Indikationen z. B. auf Patienten mit milder Herzinsuffizienz oder Vorhofflimmern, aber auch zur Rückbesinnung auf die Patienten mit breiterem QRS-Komplex und Linksschenkelblock, die am meisten profitieren. Neue Techniken wie das Conduction-System-Pacing werden die Schrittmachertherapie bei Herzinsuffizienz verändern, als Alternative oder Ergänzung zur CRT.

## Abstract (en)

The first permanent biventricular pacing system was implanted more than 30 years ago. In this article, the historical development of cardiac resynchronization therapy (CRT), starting with the pathophysiological concept, followed by the initial “proof of concept” studies and finally the large prospective-randomized studies that led to the implementation of CRT in heart failure guidelines, is outlined. Since the establishment of CRT, both an expansion of indications, e.g., for patients with mild heart failure and atrial fibrillation, but also the return to patients with broad QRS complex and left bundle branch block who benefit most of CRT has evolved. New techniques such as conduction system pacing will have major influence on pacemaker therapy in heart failure, both as an alternative or adjunct to CRT.

## Ventrikuläre Dyssynchronie durch Linksschenkelblock und rechtsventrikuläre Stimulation – Entwicklung des pathophysiologischen Konzepts

Seit der ersten Herzschrittmacherimplantation beim Menschen 1958 durch Ake Senning gab es ständig Verbesserungen der Herzschrittmachertherapie, die zum einen einer Vereinfachung des Eingriffs dienten, wie z. B. die Einführung transvenöser Sonden, zum anderen die elektrische Stimulation des Herzens dem physiologischen Kontraktionsablauf annähern sollten, wie z. B. die Einführung der 2‑Kammer-Schrittmacher 1978. Schon seit 1925 war aus experimentellen Untersuchungen bekannt, dass eine asynchrone, elektrische Stimulation des Herzens zu einer Verminderung der linksventrikulären dP/dt und Verlängerung der isovolumetrischen Kontraktionszeit führt [1]. Erst phonokardiographische und mechanokardiographische Untersuchungen durch Baragan et al. in den 1960er-Jahren konnten dann eine Verlängerung der isovolumetrischen Kontraktions- und Relaxationszeit bei Patienten mit Linksschenkelblock (LSB) nachweisen [2]. Echokardiographische und hämodynamische Untersuchungen Ende der 70er-Jahre und in den 80er-Jahren bestätigten diese negativen Effekte des LSB auf die Hämodynamik [3–5]. In der gleichen Zeit konnten ähnliche Ergebnisse auch für die AV-synchrone, rechtsventrikuläre Stimulation im Vergleich zur AAI-Stimulation gezeigt werden, als Hinweis darauf, dass eine rechtsventrikuläre Stimulation eine ähnliche Verspätung der linksventrikulären Erregung hervorruft wie ein LSB [6, 7]. Grundlegende, experimentelle Untersuchungen erklärten die negativen Effekte der ventrikulären Dyssynchronie durch regionale Veränderungen der myokardialen Last und Perfusion [8]. Allerdings richtete sich das Interesse der Elektrophysiologen zunächst noch mehr auf den Einfluss der atrioventrikulären (AV) Überleitungszeit auf die Hämodynamik als auf die Effekte der ventrikulären Dyssynchronie. Mittels Radionuklidventrikulographie konnte eine Verbesserung des Schlagvolumens bei AV-Zeiten von 150 ms gegenüber langen AV-Zeiten von 250 ms gezeigt werden [9]. Hochleitner et al. berichteten in einer kleinen, nichtrandomisierten Serie von Patienten mit dilatativer Kardiomyopathie über eine Verbesserung von NYHA-Klasse und linksventrikulärer Ejektionsfraktion bei einer rechtsventrikulären DDD-Stimulation mit einer AV-Zeit von 100 ms [10]; Brecker et al. konnten in echokardiographischen Untersuchung eine Verlängerung der ventrikulären Füllungszeit sowie eine Abnahme der Mitral- und Trikuspidalinsuffizienz sowie eine Verbesserung des „cardiac output“ bei Verkürzung der AV-Zeit zeigen [11]. Die Effekte waren jedoch nur moderat und konnten nie in randomisierten Studien bestätigt werden.

## Die ersten biventrikulären Implantationen

Die Arbeiten zu den negativen Effekten der Dyssynchronie auf die linksventrikuläre Funktion wie auch die Studien zur AV-Zeit-Optimierung mittels konventionellen DDD-Schrittmachern bei dilatativer Kardiomyopathie waren die Grundlage für die ersten Versuche, die linksventrikuläre Hämodynamik mittels biventrikulärer Stimulation zu optimieren. Allerdings waren schon akute Untersuchungen bei Patienten mit LSB unmittelbar nach Aortenklappenersatz von Gibson et al. zur biventrikulären Stimulation [12] und von de Teresa et al. [13] zur linksventrikulären Stimulation publiziert worden. Morton Mower erhielt 1987 das Patent für das Konzept der biventrikulären Stimulation. Die erste Implantation eines permanenten, biventrikulären Schrittmachers erfolgte in den Niederlanden im März 1993 durch das Team um Patricia Bakker [14]. Die Implantation der linksventrikulären Elektrode erfolgte mittels einer linksanterolateralen Mini-Thorakotomie, die Stimulation beider Ventrikel wurde durch Konnektion von 2 unipolaren Elektroden für den rechten und linken Ventrikel über ein Y‑Stück realisiert. Die Einführung der linksventrikulären Stimulation über die Äste des Sinus coronarius [15] sowie der *Over-the wire-Technik* zur Implantation [16] waren weitere, wesentliche Fortschritte, die für die Einführung in die klinische Routine von entscheidender Bedeutung waren.

## Die ersten randomisierten Studien: „proof of concept“

Ermutigt durch die ersten Erfahrungen wurden in den 90er-Jahren erste randomisierte Studien an Patienten durchgeführt. Sowohl die MUSTIC-Studie als auch die PATH-CHF-Studie waren im Cross-over-Design angelegt [17, 18]. Untersucht wurden Patienten mit fortgeschrittener Herzinsuffizienz (NYHA III–IV) und verbreitertem QRS-Komplex. Es konnte in beiden Studien eine signifikante Verbesserung der 6‑Minuten-Gehstrecke, der maximalen Sauerstoffaufnahme im kardiopulmonalen Belastungstest und der Parameter der Lebensqualität gezeigt werden. In der PATH-CHF-Studie konnte zudem erstmalig ein reverses Remodeling der linken Herzkammer unter CRT nachgewiesen werden [19]. Die Studien waren jedoch noch zu klein, um einen positiven Effekt der biventrikulären Stimulation auf *harte* Endpunkte wie Mortalität oder Hospitalisationsrate nachweisen zu können. Es ging zunächst um den Nachweis, dass das Konzept prinzipiell funktioniert („proof of concept“). Besonders in der PATH-CHF-Studie wurden auch umfangreiche, invasive Untersuchungen zur Variation von AV-Zeit und Stimulationsort auf die kardiale Hämodynamik durchgeführt. Dazu wurden intraoperativ Druckabnehmer in Aorta und linken Ventrikel gelegt. Die Ergebnisse dieser invasiven Messungen bilden immer noch die wesentliche Grundlage für unser heutiges Verständnis, wie die biventrikuläre Stimulation zu einer verbesserten Hämodynamik bei Herzinsuffizienz mit ventrikulären Leitungsstörungen führt [20]. Auch die Bezeichnung „kardiale Resynchronisationstherapie“ (CRT) stammt aus dieser Zeit. Zur gleichen Zeit konnten experimentelle Ergebnisse der Arbeitsgruppe um David Kass in den USA zeigen, dass der hämodynamische Nutzen der CRT, im Gegensatz z. B. zu medikamentösen Inotropika, nicht mit einem erhöhten Energiebedarf einherging [21]. Dies war besonders wichtig, da zu der damaligen Zeit jede positiv-inotrop wirkende Therapie von Herzinsuffizienz-Spezialisten besonders kritisch beurteilt wurde.

**Figure 1:**
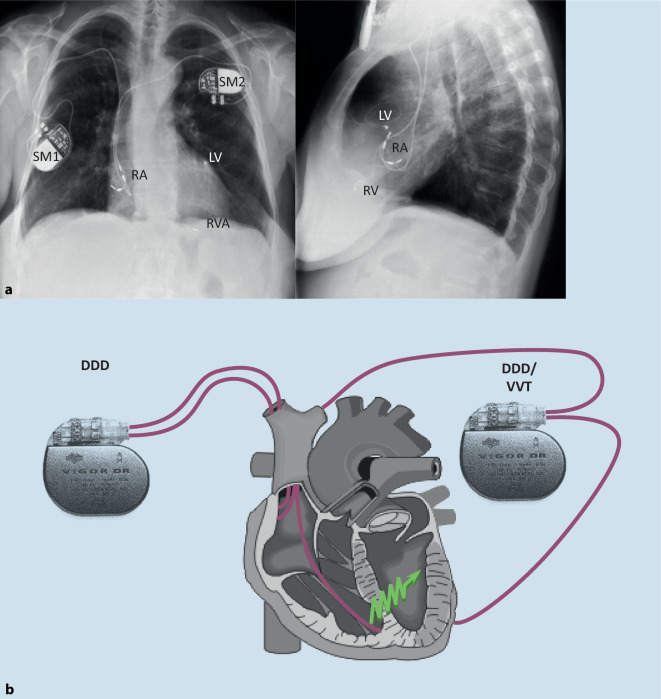

*Aus persönlicher Erinnerung sei angemerkt, dass manchmal invasive Therapien erst dann unter Kollegen akzeptiert werden, wenn man individuelle Therapie-Erfolge sieht. Wir hatten einen der ersten Patienten in Aachen im Rahmen der PATH-CHF-Studie mit einem biventrikulären Schrittmachersystem versorgt, einen 72-jährigen Patienten mit ischämischer Kardiomyopathie nach großem Vorderwandinfarkt und breitem Linksschenkelblock. Da zu diesem Zeitpunkt transvenöse Sonden noch nicht verfügbar waren, erfolgte die Implantation der linksventrikulären Elektrode noch per Mini-Thorakotomie, die Stimulation mit 2 Herzschrittmachern, wobei die linksventrikuläre Stimulation über den 2. Schrittmacher auf den rechtsventrikulären Impuls des ersten Schrittmachers getriggert erfolgte* (Abb. 1)*. Die Implantation war für die schwerkranken Patienten mit Herzinsuffizienz NYHA III–IV ein durchaus belastender Eingriff, was dazu führte, dass die Rekrutierung für die Studie schwierig war. Im Studienprotokoll war eine Aktivierung des Herzschrittmachersystems erst bei der ersten Kontrolle nach der Implantation vor Entlassung vorgesehen, so dass unmittelbar postoperativ bei dem Patienten keine CRT bestand. Als sich der Patient in der Nacht nach dem Eingriff hämodynamisch dramatisch verschlechterte und trotz hoher Dosen an Katecholaminen nur noch einen systolischen Druck von 60–70* *mm* *Hg aufbaute, wurde ich als Oberarzt durch die Intensivstation informiert. Ich entschloss mich dann, das Studienprotokoll zu verletzen und in der Nacht die biventrikuläre Stimulation zu initiieren. Unmittelbar nach der Aktivierung stieg der systolische Druck auf 100* *mm* *Hg an, die Katecholamine konnten reduziert und schon am nächsten Morgen abgesetzt werden, einen Tag später verließ der Patient die Intensivstation. Ab diesem Zeitpunkt wurde ich bei jedem Patienten mit reduzierter, linksventrikulärer Funktion und LSB gefragt, ob nicht auch eine CRT in Frage käme.*

Aus dieser Zeit stammen auch zahlreiche Untersuchungen zu den funktionellen und metabolischen Effekten der CRT. So konnte gezeigt werden, dass das bekannte Phänomen der septalen Glukose-Minderaufnahme bzw. -perfusion in szintigraphischen Untersuchungen bei LSB, hervorgerufen durch die Kontraktion des Septums gegen eine geringe Nachlast bei noch erschlaffter Lateralwand, durch CRT aufgehoben werden kann [22, 23]. Daneben konnten u. a. auch regionale Veränderungen des oxydativen Metabolismus nachgewiesen werden [24].

**Figure 2:**
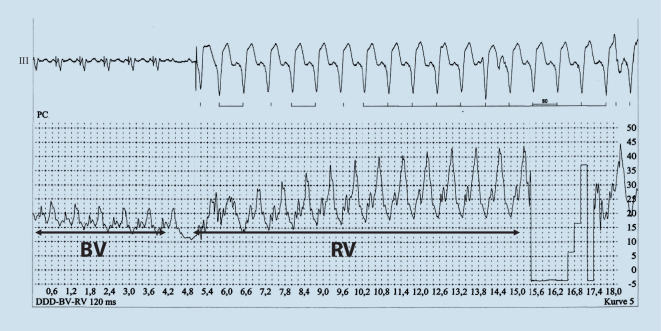

Auch konnte gezeigt werden, dass die Abnahme der v‑Welle im pulmonalkapillären Druck unter CRT durch eine Reduktion der funktionellen Mitralinsuffizienz unter CRT hervorgerufen wird ([25]; Abb. 2); auch eine zentrale Schlafapnoe bei Herzinsuffizienz kann mittels CRT reduziert werden [26].

## Der Durchbruch: COMPANION und CARE-HF

Nach den ersten, positiven Ergebnissen musste der Beweis erbracht werden, dass die Verbesserung der linksventrikulären Funktion auch zu einer Verbesserung der Mortalität und Hospitalisationsrate in prospektiv-randomisierten Studien führt. Dies gelang in der COMPANION- [27] und CARE-HF-Studie [28] für Patienten mit breitem QRS-Komplex und fortgeschrittener Herzinsuffizienz (NYHA-Klasse III–IV). Während CARE-HF in einem 2‑armigen Design eine optimale, medikamentöse Herzinsuffizienztherapie mit einem zusätzlichen, biventrikulären Herzschrittmacher (CRT-P) verglich, wurde in der COMPANION-Studie auch ein dritter Studienarm mit kombinierter CRT- und ICD-Therapie (CRT-D) untersucht. In beiden Studien kam es zu einer signifikanten Senkung des kombinierten Endpunkts aus Mortalität und Hospitalisationen durch die CRT, ein signifikanter Überlebensvorteil für CRT‑D vs. CRT‑P konnte in COMPANION nicht gezeigt werden – eine Frage, die bis heute nicht durch randomisierte Studien geklärt ist. Allerdings war die Studie auch von der Stichprobengröße nicht geeignet, diese Frage zu beantworten.

Die besondere Bedeutung von COMPANION und CARE-HF lag darin, dass die positiven Ergebnisse dieser Studien erstmalig zur Aufnahme der CRT in die Herzinsuffizienz-Leitlinien führten. Allerdings waren beide Studien auf Patienten im Sinusrhythmus und mit fortgeschrittener Herzinsuffizienz (NYHA III–IV) beschränkt, und so kam eine CRT nur für ca. 7–10 % aller Herzinsuffizienzpatienten in Frage [29, 30]. Es stellte sich also die Frage, ob auch Patienten mit milder Herzinsuffizienz und Vorhofflimmern profitieren.

## Indikationsausweitung: milde Herzinsuffizienz, Herzschrittmacher-Upgrade, Vorhofflimmern

### *Herzinsuffizienz NYHA I–II*.

Drei Studien untersuchten die Frage, ob CRT bei milder Herzinsuffizienz zu einer verzögerten Progression der Erkrankung und Mortalitätssenkung führt: REVERSE [31], MADIT-CRT [32] und RAFT [33]. In der REVERSE-Studie wurden 610 Patienten mit primärpräventiver ICD-Indikation und Herzinsuffizienz Klasse NYHA I–II auf aktive vs. inaktive CRT randomisiert; durch CRT kam es zu einer Verbesserung der Ejektionsfraktion und Hospitalisationsrate, eine Verbesserung von Mortalität, Lebensqualität und 6‑Minuten-Gehstrecke konnte jedoch nicht gezeigt werden. Diese gelang in der größeren MADIT-CRT-Studie, in der 1820 Patienten der NYHA-Klasse I–II und mit primärpräventiver ICD-Indikation auf CRT‑D vs. ICD randomisiert wurden. In dieser Studie konnte erstmalig eine signifikante Verbesserung des kombinierten Endpunkts von Überlebens- und Hospitalisationsrate durch CRT bei milder Herzinsuffizienz gezeigt werden – allerdings war der kombinierte Endpunkt fast ausschließlich durch die Hospitalisationsrate getrieben, die Mortalität wurde durch CRT nicht signifikant gesenkt. Der Nachweis einer Mortalitätssenkung gelang in der RAFT-Studie, die allerdings Patienten mit etwas fortgeschrittener Herzinsuffizienz untersuchte: Es wurden 1798 Patienten mit QRS-Breite > 120 ms, Indikation für einen ICD und Herzinsuffizienz NYHA II-III auf ICD vs. CRT‑D randomisiert. In diesem Kollektiv kam es nach 40 Monaten mittlerer Nachbeobachtung auch zu einer signifikanten Senkung der Mortalität um 25 %. Man konnte also folgern, dass auch bei milder Herzinsuffizienz ein Nutzen der CRT besteht. Wenngleich bei NYHA I–II ein Mortalitätsvorteil in MADIT-CRT und REVERSE nicht nachweisbar war, so konnte doch auch für diese Gruppen ein reverses Remodeling und eine Senkung der Hospitalisationsrate gezeigt werden. Daher wurde in den folgenden Leitlinien sowohl der ESC als auch des American College of Cardiology (ACC) bzw. der American Heart Association (AHA) die CRT-Indikation auch auf die NYHA-Klasse II erweitert, allerdings nicht auf die NYHA-Klasse I, denn in den Studien waren nur relativ wenig NYHA-I-Patienten eingeschlossen worden.

### *Herzschrittmacher-Upgrade*.

Seit Einführung der Herzschrittmachertherapie (s. Kapitel „Geschichte der Herzschrittmachertherapie“) war der routinemäßig verwendete, ventrikuläre Stimulationsort die rechtsventrikuläre (RV) Spitze, vor allem auf Grund der Einfachheit der Sondenplatzierung. Erst seit Mitte der 80er-Jahre wiesen experimentelle und echokardiographische Untersuchungen darauf hin, dass die apikale RV-Stimulation einen ähnlich negativen, hämodynamischen Effekt induziert wie ein spontaner LSB [6, 7]. Schließlich konnte Anfang des Jahrtausends in den Studien DAVID [34] und MOST [35] gezeigt werden, dass es bei > 40 % RV-Stimulation bei 2‑Kammer-Schrittmachern oder -ICD zu einer Erhöhung der Hospitalisationsrate wegen Herzinsuffizienz im Verlauf kommt, womit die Grundlage für die Hypothese gelegt war, dass CRT bei Patienten mit linksventrikulärer Dysfunktion und Notwendigkeit der ventrikulären Stimulation indiziert sein könnte. Dies wurde in der BLOCK-HF-Studie [36] untersucht, in die 691 Patienten mit Herzinsuffizienz NYHA I–III, linksventrikulärer Ejektionsfraktion (LVEF) < 50 % und Indikation für einen Herzschrittmacher wegen höhergradigem AV-Block auf konventionelle DDD-Stimulation vs. CRT randomisiert wurden. Der kombinierte Endpunkt aus Tod, Hospitalisation wegen Herzinsuffizienz und Zunahme des linksventrikulären, endystolischen Volumenindex um > 15 % wurde durch die CRT signifikant reduziert, allerdings fast ausschließlich durch die geringere Volumenzunahme bedingt. Eine weitere Studie, die BIOPACE-Studie [37], welche die Patienten unabhängig von der LVEF auf konventionelle DDD- vs. CRT-Stimulation randomisierte, konnte keinen signifikanten Nutzen der CRT belegen; die Studie wurde lediglich als Hotline-Vortrag auf dem Europäischen Kardiologenkongress 2014 präsentiert, aber nie endgültig publiziert. In einer retrospektiven Langzeitanalyse konnten Kiehl et al. [38] zeigen, dass es nur bei ca. 10 % aller Patienten mit normaler LV-Funktion unter konventioneller DDD-Stimulation zu einer Verschlechterung der linksventrikulären Funktion kam, in Abhängigkeit vom rechtsventrikulären Stimulationsanteil. In einigen, kleineren Studien ergaben sich Hinweise, dass dieses Remodeling durch CRT verhindert werden kann [39, 40]. In den aktuell gültigen Leitlinien der ESC zur Herzschrittmachertherapie wird die Indikation zur CRT bei primärer, antibradykarder Indikation erst ab einer LVEF < 40 % gesehen [41]. Sollte sich bei einem Schrittmacher- oder ICD-Patienten im Verlauf eine Herzinsuffizienz entwickeln, kann ein Upgrade auf eine CRT sinnvoll sein. In Europa sind aktuell etwa ein Viertel aller CRT-Implantationen Upgrade-Eingriffe [42]. Dieses Konzept konnte erst vor Kurzem in der BUDAPEST-Studie [43] belegt werden: 360 Patienten mit implantiertem Herzschrittmacher oder ICD, Herzinsuffizienz NYHA II–IV, ≥ 20 % RV-Stimulation und einer stimulierten QRS-Breite wurden 3:2 auf CRT‑D oder ICD randomisiert. Nach etwas mehr als einem Jahr kam es in der CRT‑D Gruppe signifikant seltener zu Todesfällen und Hospitalisationen. In den aktuellen Leitlinien besteht eine Klasse-IIa-Indikation für Patienten mit einer symptomatischen Herzinsuffizienz, einer LVEF < 35 % und > 20 % RV-Stimulation [41].

### *Vorhofflimmern*.

Die meisten der o. g. prospektiv-randomisierten Studien zur CRT haben nur Patienten im Sinusrhythmus eingeschlossen, nur in der RAFT-Studie waren auch wenige Patienten mit Vorhofflimmern. Die Implantationspraxis in Europa zeigt aber, dass ca. 40 % aller Patienten, die ein CRT-System erhalten, entweder persistierendes oder paroxysmales Vorhofflimmern haben [44]. Rasch zeigte sich, dass für eine effektive CRT eine Kontrolle der AV-Knoten-Überleitung erforderlich ist. Ob dazu regelhaft eine AV-Knoten-Ablation erforderlich ist, ist bis heute nicht endgültig geklärt. Eine große Register-Analyse von Gasparini et al. [45] weist auf eine Überlegenheit der AV-Knoten-Ablation hin; andere Autoren konnten allerdings zeigen, dass eine medikamentöse Frequenzkontrolle, wenn eine biventrikuläre Stimulationsrate von mindestens 94 % erreicht werden kann, nicht unterlegen ist [46]. Die ESC-Leitlinie [41] empfiehlt heute die Implantation eines CRT-Systems bei Vorhofflimmern erst ab NYHA-Klasse III und bei LVEF < 35 %. Eine besondere Gruppe sind Patienten mit schnell übergeleitetem Vorhofflimmern, bei denen eine Vorhofflimmerablation nicht möglich oder vom Patienten nicht erwünscht ist und die daher einer AV-Knoten-Ablation zugeführt werden. Für diese konnte in den Studien APAF [47] und APAF-CRT [48] eine Verbesserung von Mortalität, Hospitalisationsrate und Lebensqualität durch eine CRT vs. konventionelle RV-Stimulation gezeigt werden. Die ESC-Leitlinie [41] spricht auf Grund dieser Daten eine Klasse-IIa-Empfehlung für die CRT bei mindestens moderat reduzierter, linksventrikulärer Funktion aus.

## Rückbesinnung: Nicht jede QRS-Verbreiterung ist gleich und das Problem der echokardiographischen Asynchronie

In den ersten, großen randomisierten CRT-Studien COMPANION und CARE-HF wurde kaum zwischen den verschiedenen Formen der QRS-Verbreiterung unterschieden, so dass im Prinzip jede QRS-Verbreiterung, LSB, RSB und auch atypische Formen für eine CRT in Frage kamen. Aus den o. g. pathophysiologischen Erwägungen war ein wesentlicher Nutzen der CRT aber vor allem bei LSB zu erwarten. Die ersten, deutschen Leitlinien zur CRT trugen dieser Vorstellung Rechnung, indem bei Nicht-LSB-Patienten lediglich eine Klasse-IIb-Indikation zur CRT zugelassen wurde [49]. Eine solche Unterscheidung nach Art der QRS-Verbreiterung war in den Leitlinien von ESC und ACC/AHA zunächst nicht zu finden. Erst in der MADIT-CRT-Studie fand sich überhaupt eine prospektiv angewandte Definition der Blockbilder bei Patienteneinschluss, was eine Analyse des Nutzens der CRT in Abhängigkeit von der Art der QRS-Verbreiterung möglich machte. Die Daten bestätigten, dass der Nutzen v. a. bei LSB vorhanden ist, weniger bei RSB oder unspezifischer QRS-Verbreiterung [50]. Daraufhin wurde die CRT-Indikation auch in den internationalen Leitlinien differenzierter eingeteilt.

Auch gab es Versuche, Patienten mit schmalem QRS, aber echokardiographisch nachweisbarer Asynchronie mittels CRT zu optimieren. Nachdem einige, kleinere Studien einen potenziellen Nutzen eines solchen Vorgehens nahelegten [51–53], konnte dies in 2 prospektiven Studien nicht bestätigt werden [54, 55]. Die Echo-CRT-Studie wies sogar auf eine potenzielle Übersterblichkeit bei Patienten mit einem QRS-Komplex < 130 ms hin, was zu einer Anpassung in den Leitlinien zur CRT führte. Die Ergebnisse dieser Studien haben wesentlich dazu beigetragen, dass bis heute die Echokardiographie für die Patientenauswahl zur CRT über die Bestimmung der LVEF hinaus keine wesentliche Rolle spielt, obwohl die CRT die mechanische Dyssynchronie bei ventrikulären Leitungsstörungen korrigieren soll, die mit der Echokardiographie am besten bzw. einfachsten erfasst werden kann. Eine aktuelle Studie, die AMEND-CRT-Studie [56], untersucht derzeit, ob neuere, echokardiographische Parameter der Dyssynchronie zusätzlich zu den EKG-Kriterien möglicherweise doch eine genauere Identifikation geeigneter CRT-Kandidaten ermöglichen, auch weil die Non-Responder-Rate der CRT immer noch hoch ist.

Auch die Bedeutung bildgebender Verfahren zur optimierten Platzierung der linksventrikulären Elektrode ist nicht endgültig geklärt. Während einige Studien einen Nutzen echokardiographischer Verfahren zeigten [57, 58], konnte dies in anderen Studien nicht bestätigt werden [59–61].

### *Non-Responder*.

Die Non-Responder-Rate ist bis heute ein immer noch nicht vollständig gelöstes Problem. Etwa ein Drittel der Patienten zeigt keine akute, hämodynamische Verbesserung unter CRT [62]. Allerdings ist die Definition des Non-Responders in der Literatur uneinheitlich. Neben der akuten, hämodynamischen Antwort, die in der klinischen Routine heute kaum noch gemessen wird, kann man Response als Volumen-Response (reverses Remodeling unter CRT) oder symptomatische Besserung (z. B. Verbesserung der NYHA-Klasse) definieren. Legt man Letzteres zugrunde, gibt es auch bei medikamentöser Herzinsuffizienztherapie eine vergleichbare Non-Responder-Rate, z. B. für Enalapril [63] oder β‑Blocker [64]. Zudem sind symptomatische Besserung und reverses Remodeling nicht gut korreliert. Vor allem Patienten mit hoher Narbenlast können hämodynamisch und auch symptomatisch profitieren, zeigen aber weniger reverses Remodeling. Die Ursachen für eine fehlende Verbesserung unter CRT sind mannigfaltig (Tab. 1) und nur z. T. durch eine optimierte Implantationstechnik bzw. postoperative Programmierung korrigierbar. Untersuchungen zur Nachsorge nach CRT zeigen, dass oft auch die medikamentöse Therapie nicht ausreichend optimiert ist [65]. Dies weist auf die Notwendigkeit der engen Kommunikation zwischen Herzinsuffizienzspezialisten und Elektrophysiologen auch nach der Implantation der Systeme hin.

**Table 1:** 

Präoperativ – Hohe Narbenlast bzw. posterolaterale Narbe – Extreme Dyssynchronie – Schmaler QRS (< 130 ms) – QRS-Verbreiterung ohne typische LSB-Konfiguration – Extreme LV-Dilatation – Pulmonale Hypertonie – Ausgeprägte RV-Dysfunktion – Fortgeschrittene Niereninsuffizienz – Relevantes Klappenvitium (außer funktionelle Mitralinsuffizienz)
Intraoperativ – Ungünstige Lage der LV-Elektrode (z. B. anterior)
Postoperativ – Fehlende medikamentöse Therapie-Optimierung – Zahlreiche VES – Vorhofflimmern mit unzureichender Frequenzkontrolle – Fortschreiten der Grunderkrankung

## Ausblick: Conduction-System-Pacing

Trotz der unbestreitbaren Erfolge der CRT gibt es neben der Non-Responder-Rate auch noch einige andere Probleme, wie z. B. eine ungeeignete Koronarvenenanatomie für die Implantation der linksventrikulären Elektrode, eine Stimulation des linksseitigen Nervus phrenicus, wenn dieser in anatomischer Nähe zum Stimulationsort im Koronarsinus liegt, und die epikardiale Stimulation, für die in experimentellen Untersuchungen ein proarrhythmogener Effekt gezeigt wurde [66]. In jüngerer Zeit wurde die selektive Stimulation des spezifischen Reizleitungssystems zur hämodynamischen Optimierung der Schrittmachertherapie für Patienten mit AV-Überleitungsstörungen, aber auch für CRT-Kandidaten entwickelt (s. a. Artikel Lemke). Die Stimulation des His-Bündels vermag dabei im Gegensatz zur konventionellen, rechtsventrikulären Stimulation z. B. in der Ventrikelspitze eine physiologischere, ventrikuläre Erregungsausbreitung herzustellen. Allerdings ist die His-Bündel-Stimulation durch hohe Reizschwellen und eine vergleichsweise hohe Dislokationsrate der His-Bündel-Elektroden limitiert, weswegen bei Patienten mit höhergradigem AV-Block die Platzierung einer rechtsventrikulären Backup-Elektrode empfohlen wird. Zusätzlich konnte in der His-SYNC-Studie gezeigt werden, dass in fast 50 % der Fälle eine Aktivierung des distalen Reizleitungssystems nicht gelang und daher ein Crossover zur biventrikulären Stimulation erforderlich war [67]. In einer neueren Studie waren beide Ansätze ähnlich effektiv, die Reizschwellen bei His-Bündel-Stimulation allerdings höher [68]. Daher wird statt der His-Bündel-Stimulation inzwischen zunehmend die Stimulation des linken Schenkels bzw. des umgebenden Areals (sog. „left bundle branch area pacing“, LBBAP) eingesetzt. Die Implantationstechnik für LBBAP ist einfacher, die Sonden werden endokardial platziert, die Reizschwellen sind niedriger und die Dislokationsrate auf Grund der Fixierung der Elektroden im Septum kein klinisches Problem mehr. In mehreren Observationsstudien war die stimulierte QRS-Breite bei LBBAP schmaler als unter CRT, die Verbesserung der LVEF stärker und Hospitalisationsrate im Verlauf geringer [69–72]. Bisher ist nur eine kleine, prospektiv-randomisierte Studie zu LBBAP vs. CRT an 40 Patienten mit LSB und nichtischämischer Kardiomyopathie publiziert [73]; auch hier zeigte sich eine stärkere Verbesserung der LVEF sowie Abnahme der LV-Volumina unter LBBAP, allerdings kein relevanter Unterschied im Hinblick auf die funktionelle Verbesserung. Es sind noch weitere Daten an größeren Patientenzahlen und mit längerer Nachbeobachtung erforderlich, um den Stellenwert des LBBAP im Vergleich zur CRT endgültig zu beurteilen. Mehrere prospektiv-randomisierte Studien laufen derzeit. Schon jetzt ist LBBAP aber eine sinnvolle Alternative, wenn z. B. eine LV-Sondenplatzierung nicht möglich ist [74]. Auch gibt es Hinweise, dass die Kombination einer LV-Elektrode mit LBBAP oder His-Bündel-Pacing den Effekt der CRT weiter verbessern kann [75].
